# Levels of knowledge regarding malaria causes, symptoms, and prevention measures among Malawian women of reproductive age

**DOI:** 10.1186/s12936-020-03294-6

**Published:** 2020-06-24

**Authors:** Alick Sixpence, Owen Nkoka, Gowokani C. Chirwa, Edith B. Milanzi, Charles Mangani, Don P. Mathanga, Peter A. M. Ntenda

**Affiliations:** 1 Malaria Alert Centre (MAC), College of Medicine (CoM), University of Malawi (Unima), Private Bag 360, Chichiri, Blantyre 3, Malawi; 2 School of Public Health (SPH), Taipei Medical University (TMU), No. 250, Wuxing Street, Xinyi District, Taipei City, 110 Taiwan; 3 Institute for Health Research and Communication (IHRC), P.O Box 1958, Lilongwe, Malawi; 4 Department of Economics, Chancellor College (Chanco), University of Malawi (Unima), P.O. Box 280, Zomba, Malawi; 5 MRC Clinical Trials Unit at UCL, Institute of Clinical Trials & Methodology, 90 High Holborn 2nd Floor, London, WC1V 6LJ UK; 6 Department of Public Health, School of Public Health and Family Medicine (SPHFM), University of Malawi (Unima), Private Bag 360, Chichiri, Blantyre 3, Malawi

**Keywords:** Malaria, Malaria-related knowledge, Multinomial logistic, Knowledge scores, Malawi

## Abstract

**Background:**

Malawi is a malaria-endemic country and approximately 6 million cases are reported annually. Improving knowledge of malaria causes and symptoms, and the overall perception towards malaria and its preventive measures is vital for malaria control. The current study investigated the levels of knowledge of the causes, symptoms and prevention of malaria among Malawian women.

**Methods:**

Data from the 2017 wave of the Malawi Malaria Indicator Survey (MMIS) were analysed. In total, 3422 women of reproductive age (15–49 years) were sampled and analysed. The levels of women’s knowledge about: (1) causes of malaria; (2) symptoms of malaria; and, (3) preventive measures were assessed. The tertiles of the composite score were used as the cut-offs to categorize the levels of knowledge as ‘low’, ‘medium’ and ‘high’. Multinomial logistic regression models were constructed to assess the independent factors while taking into account the complex survey design.

**Results:**

Approximately 50% of all respondents had high levels of knowledge of causes, symptoms and preventive measures. The high level of knowledge was 45% for rural women and 55% for urban dwellers. After adjusting for the a wide range of factors, women of age group 15–19 years adjusted odds ratio ((aOR): 2.58; 95% Confidence Interval (CI) 1.69–3.92), women with no formal education (aOR: 3.73; 95% CI 2.20–6.33), women whose household had no television (aOR: 1.50; 95% CI 1.02–2.22), women who had not seen/heard malaria message (aOR: 1.53; 95% CI 1.20–1.95), women of Yao tribe (aOR: 1.95; 95% CI 1.10–3.46), and women from rural areas had low levels of knowledge about the causes of malaria, symptoms of malaria and preventive measures. Additionally, the results also showed that women aged 15–19 years (beta [β] = − 0.73, standard error [SE] = 0.12); *P *< .0001, women with no formal education (β = − 1.17, SE = 0.15); *P *< .0001, women whose household had no radio (β = − 0.15, SE = 0.0816); *P *= 0.0715 and women who had not seen or heard malaria message (β = − 0.41, SE = 0.07); *P *< .0001 were likely to have a lower knowledge score.

**Conclusions:**

The levels of malaria knowledge were reported to be unsatisfactory among adult women, underscoring the need to scale up efforts on malaria education. Beside insecticide-treated bed nets (ITNs) and prompt diagnosis, malaria can be best managed in Malawi by increasing knowledge of malaria causes, and symptoms especially for younger women, women with no formal education, women whose households have no media, women from Yao tribes, and rural dwellers.

## Background

Malaria, a life-threatening disease caused by *Plasmodium* parasites, is a major public health issue in many tropical and sub-tropical areas, especially in sub-Saharan Africa (SSA) countries [1, 2]. The World Health Organization (WHO) estimates that 228 million cases and 405,000 malaria-related deaths occurred in 2018 [2–4]. The SSA region bears an excessively high portion of the global malaria problem. By the end of 2017, the sub-Saharan region was home to 92% of malaria cases and 93% of malaria deaths [2].

In Malawi, the Ministry of Health (MoH) through the national malaria control programme (NMCP) focuses on scaling up the use of insecticide-treated bed nets (ITNs) by increasing access and ownership [5]. The programme also includes the provision of artemisinin-based combination therapy (ACT) and intermittent preventive treatment for pregnant women (IPTp) using sulfadoxine-pyrimethamine (SP), as well as early diagnosis and prompt treatment [5]. Furthermore, the NMCP has also put in place methods for vector control, such as scaling up of indoor residual spraying (IRS) to all target districts, implementation of larval source and environmental management, increased resistance monitoring and implementation of a resistance management, collection of entomological data and others [6]. Additionally, the NMCP has introduced two new interventions for vector and malaria control, which include new formulation of bed nets (Piperonyl Butoxide (PBO nets)) that are designed to manage insecticide resistance as well as RTS,S/ASO1 malaria vaccination for young children [7]. However, despite all these interventions, Malawi remains a malaria-endemic country with an estimated 6 million cases reported annually [8]. This signifies that beyond strategies and interventions to fight against malaria, human behaviour, including knowledge, can also play a vital role in reducing malaria transmission and infection.

Knowledge related to health is of great essence because consistent evidence has demonstrated the connection between individual healthy knowledge, health behaviour and health outcomes [9]. It has been reported that people with low levels of individual health knowledge are greater than two times more likely to experience poor health outcomes [10]. Unfortunately, there are inconsistent and conflicting reports regarding the levels of malaria knowledge and associated factors worldwide [11–14]. Previous studies have reported that women who reside in urban areas, women with better family monthly income, women who attended formal education have better knowledge regarding causes, signs and symptoms and preventive measures of malaria [12]. WHO reported that having a good knowledge regarding malaria causes, signs and symptoms, mode of transmission and preventive measures led to the use of malaria prevention strategies and improved health-seeking behaviour [15].

Across African countries, previous studies on malaria knowledge, attitudes and practices (KAP) have reported the influence of malaria misconceptions on malaria control efforts [11, 16–18]. Thus, evaluating malaria knowledge among women of reproductive age is of great essence since women are the ones largely involved in home-based management of malaria especially to the highly vulnerable group (children below the age of 5 years) to malaria [19]. Furthermore, in settings where malaria transmission is high (such as Malawi), women of reproductive age are also a vulnerable group and susceptible to malaria in the course of pregnancy [20, 21]. Studies reported that malaria parasites may be present in the placenta contributing to placental malaria and maternal anaemia which may result in adverse birth outcomes, such as miscarriage, stillbirth, preterm birth (PTB), low birth weight (LBW) [22].

Despite the benefits of malaria knowledge on malaria control efforts, little is known about the levels of knowledge on malaria causes, symptoms and prevention among Malawian women. Moreover, elsewhere previous studies that have examined factors linked to malaria-related knowledge have either examined the level of malaria knowledge in general (low *vs* high) or individual knowledge domains [23, 24]. However, several factors may play different roles in influencing a specific type of malaria knowledge at different levels (i.e., low, medium and high) and therefore, evaluating them separately may provide a better understanding of the factors associated with the different levels of malaria knowledge. In Malawi, no study has ever explored the factors associated with knowledge related to causes, symptoms and prevention measures of malaria using three level outcome. Moreover, those that have examined knowledge-related malaria among Malawian women have restricted the study to specific districts [25]. Using nationally representative data, this study investigated the levels of malaria knowledge on the causes, symptoms and prevention among Malawian women.

## Methods

### Study design

This was a cross-sectional study that used data from the 2017 Malawi Malaria Indicator Survey (2017 MMIS) [26]. The 2017 MMIS was conducted with respect to Roll Back Malaria Monitoring and Evaluation Reference Group (RBM-MERG) guidelines [26].

### Sampling technique

The survey used a two-stage sampling design and it permits the estimation of key malaria indicators for the country as a whole, as well as separately for urban and rural areas in all three administrative regions in Malawi. In brief, the first stage involved selecting clusters from the enumeration areas (EAs). A total of 150 clusters were selected (60 clusters in urban areas and 90 clusters in rural areas), with probability proportional to size, from the EAs covered in the 2008 Population and Housing Census (PHC). The second stage systematically selected households from a list that was undertaken in all selected EAs and households were randomly selected from these lists.

### Data collection

**Fig. 1:**
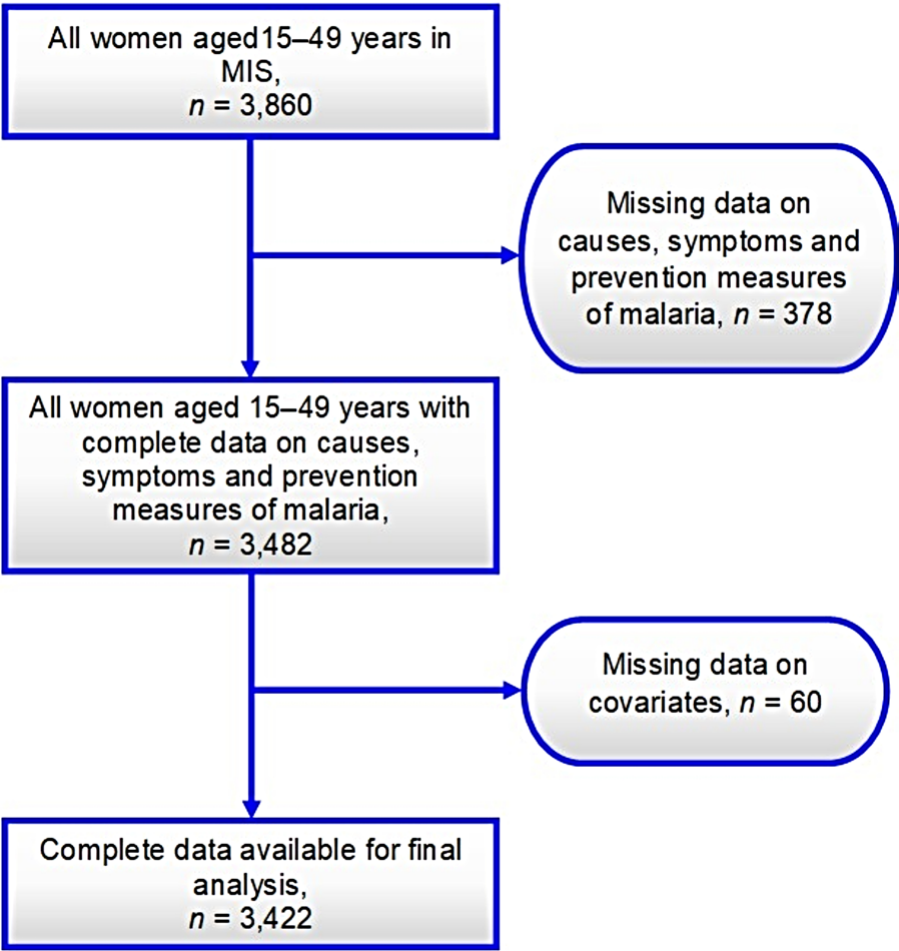
Shows the sample selection procedure

The primary objective of the 2017 MMIS was to collect information on mosquito nets, IPTp, care-seeking behaviour, and treatment of fever in children. All women were between 15 and 49 years of age who were either permanent residents of the selected households or visitors who stayed in the household the night before the survey were interviewed. Through face-to-face interview data on knowledge related to malaria were collected from the women of reproductive age. Data were primarily collected using three types of questionnaires: the Household Questionnaire, the Women’s Questionnaire, and the Biomarker Questionnaire. This study focused on responses from the women’s questionnaire, which collected malaria information (such as malaria KAP) from women aged 15–49 years. Originally, the dataset had 3860 responses from women of reproductive age. After applying inclusion and exclusion the final sample was 3422 women. The sample selection process is shown in Fig. 1.

### Study variables

#### Dependent variable

Overall, the knowledge score related to malaria was assessed by aggregating the three domains of knowledge-related variables, namely (i) causes; (ii) symptoms; and, (iii) preventive measures. The Malawi MIS 2018 included a number of basic questions that were used to explore malaria-related knowledge among adult women. These domains are described in detail as follows:

#### Knowledge about causes of malaria

Question was asked, what do you think is the cause of malaria? The following were answers: (1) mosquito bites; (2) eating immature sugarcane; (3) eating cold nsima; (4) insufficient sleeping; (5) eating dirty food; (6) drinking dirty water; (7) getting soaked in rain; and, 8) cold or changing weather.

#### Knowledge about common symptoms of malaria

Question was asked: what signs or symptoms would lead you to think that a person has malaria? The answers to this question were: (1) fever; (2) feeling cold; (3) headache; (4) vomiting; (5) diarrhoea; (6) dizziness; (7) loss of appetite; (8) body ache or joint pain; (9) pale eyes; (10) salty-tasting palms; (11) feeling weak; and, (12) refusing to eat or drink.

#### Knowledge about preventive/protective measures

Question was asked: how can someone protect themselves against malaria? The following answers were provided to the question: (1) sleep under a treated net; (2) sleeping under an insecticide-treated mosquito net; (3) using mosquito net; (4) taking preventive medication; (5) spraying the house/rooms with insecticide; (6) clear weeds around the house; (7) using mosquito coils; (8) cutting grass around house; (9) filling in stagnant waters (puddles); (10) keeping the surroundings clean; (11) burning leaves; (12) avoiding drinking dirty water; 13) avoiding eating bad food; (14) putting screens on windows; and, (15) avoid getting soaked in rain.

Comprehensive questions asked to calculate malaria knowledge score can be accessed from the MMIS report [26]. All the knowledge-related variables were recoded to binary level such that the correct answer was coded 1 while an incorrect answer was coded 0. Using an array command in SAS, knowledge score was calculated by summing up all the knowledge variables, with 0 recorded as the least possible score and 35 recorded as the highest possible score. Increasing score indicated better malaria knowledge. Finally, the tertiles of the composite score was used as the cut-off to categorize the levels of knowledge as ‘low’, ‘medium’ and ‘high’. Women who scored 33% and below of knowledge score were categorized as having low knowledge, those who scored between 34 and 66% of the knowledge score were classified as having medium knowledge, and women who scored within the exact 67% cut-off and above were classified as having high knowledge regarding causes, symptoms and prevention measures of malaria.

### Independent variables

Based on insights from relevant literature [23, 24], the following independent factors were treated appropriate for analysis: women’s age in years (15–19, 20–24, 25–29, 30–34, 35–39, 45–49); educational attainment (no formal education, primary, secondary and higher education); household has radio (no, yes); household has television (no, yes); gender of household head (male, female); wealth index (poorest/poorer/middle, richer, richest); seen/heard malaria message (no, yes); ethnicity (Chewa, Tumbuka, Lomwe, Yao, Ngoni, other); place of residence (urban, rural); and, geographical region (northern, central, southern). The wealth index is a composite measure of a household’s cumulative living standard. The household wealth index is derived using easy-to-collect data on a household’s ownership of selected assets, such as televisions and bicycles, materials used for housing construction and types of water access and sanitation facilities [27]. Using a statistical procedure known as principal component analysis (PCA), the wealth index assigned individual households on a continuous scale of relative wealth.

### Statistical analyses

Firstly, descriptive analyses were conducted whereby the baseline characteristics of the study sample were presented as weighted frequencies and percentages. Secondly, the bivariate analyses were performed using Chi square (Rao-Scott Chi Square Test) to determine the distribution of sociodemographic characteristics according to the levels (low, medium, high) of knowledge related to causes, symptoms and prevention of malaria. The variables that indicated significance at *P* value equal or less than 0.25 on Chi square were retained for regression analysis in order to allow the inclusion of more relevant descriptive variables. Thirdly, the multivariate analyses were performed by multinomial logistic regression models with the three-level outcome variable, low, medium and high levels of knowledge related to causes, symptoms and prevention of malaria. Furthermore, multiple linear regression model was used to investigate the factors associated with knowledge related to malaria among adult women (i.e., knowledge score was examined as a continuous variable). Due to complex design of the sampling technique (two-stage cluster), the survey-specific SAS procedures for weighting, clustering and stratification in the survey design were conducted. In each model, adjusted odds ratios (aORs) and 95% confidence intervals (CIs) with their p-values were calculated. The results of the multiple linear regression were expressed as standardized betas and standard errors. Variance Inflation Factor (VIF) and tolerance were conducted to examine the presence of multicollinearity among the independent factors at the cut-offs of 10 and greater than 0.1. Significance was defined as *p*-value < 0.05. All data analyses were conducted using SAS for Windows, version 9.4 (SAS Institute, Cary, NC, USA).

### Ethical consideration

The 2017 MMIS data are available for public use upon request from the Measure Demographic and Health Survey (Measure DHS Programme). The 2017 MMIS was implemented by the Malawi National Malaria Control Programme (NMCP) while the ICF offered technical assistance through the DHS Programme. The 2017 MMIS received ethical clearance from the Malawi National Health Sciences Research Committee (NHSRC) and the Institutional Review Board (IRB) of ICF Macro. An IRB of ICF Macro ensured that the 2017 MMIS was conducted in line with the US Department of Health and Human Services regulations for the protection of human subjects—the Code of Federal Regulations Title 45: Public Welfare, part 46 (45 CFR 46) [28], while the NHSRC certified that the survey was executed in line with Malawian laws and norms. Furthermore, before the interviews, verbal informed consent was obtained from the heads of the household to participate in the household questionnaire, and each eligible woman to participate in the women’s questionnaire. Participants were assured that participation in 2017 MMIS was voluntary.

## Results

### Baseline characteristics of the study participants by place of residence

Table 1 shows the descriptive characteristics of the study participants stratified by place of residence. In total, 3422 women of reproductive age: 15–49 years (1602 women from urban and 1820 women from rural areas) were analysed in this study. Overall, approximately 50% of adult women were estimated to have the high levels of knowledge related to the causes, signs and symptoms and preventive measures of malaria. More than half of the women had primary school education (54%). However, majority of women had no radio (53%), television (72%), and had not seen/heard malaria message (64%). A majority of participants were living in male-headed households (73%) and a majority of women were southern region (54%) dwellers. The differences between women from urban and rural areas were statistically significant with respect to the women’s age (*P *= 0.0003), maternal education levels (*P *< .0001), and household having a radio (*P *< .0001), household having a television (*P *< .0001), gender of household head (*P *= 0.0367), household wealth (*P*.0001), ethnicity (*P *= 0.0003), and access to malaria messages (*P *< .0001).

**Table 1:** Characteristics of study respondents according to place of residence *n *= 3422. MMIS 2017

	Total sample	Urban	Rural	*P*-value
	*n* (%)	*n* (%)	*n* (%)	
Age of the respondents				0.0003
15–19	703 (20.5)	315 (44.8)	388 (59.2)	
20–24	694 (20.3)	340 (49.0)	354 (51.0)	
25–29	590 (17.2)	284 (48.1)	306 (51.9)	
30–34	575 (16.8)	293 (51.0)	282 (49.0)	
35–39	436 (12.7)	212 (48.6)	224 (51.4)	
40–49	424 (12.3)	158 (37.3)	266 (62.7)	
Highest educational level				<.0001
No formal education	252 (7.4)	52 (20.6)	200 (79.4)	
Primary	1847 (54.0)	609 (33.0)	1238 (67.0)	
Secondary and above	1323 (38.6)	941 (71.1)	382 (28.9)	
Household has radio				<.0001
No	1805 (52.8)	564 (31.3)	1241 (68.7)	
Yes	1617 (47.2)	1038 (64.2)	579 (35.8)	
Household has television				<.0001
No	2442 (71.4)	768 (31.5)	1674 (68.5)	
Yes	980 (28.6)	834 (85.1)	146 (14.9)	
Sex of household head				0.0367
Male	2514 (73.5)	1150 (45.7)	1364 (54.3)	
Female	908 (26.5)	452 (49.8)	456 (50.2)	
Wealth index				<.0001
Poorest/Poorer/Middle	1169 (34.2)	42 (3.6)	1127 (96.4)	
Richer	627 (18.3)	187 (29.8)	440 (70.2)	
Richest	1626 (47.5)	1373 (84.4)	253 (15.6)	
Seen/heard malaria message				0.0003
No	2200 (64.3)	979 (44.50)	1221 (55.50)	
Yes	1222 (35.7)	623 (50.98)	599 (49.02)	
Ethnicity				<.0001
Chewa	977 (28.5)	426 (43.6)	551 (56.4)	
Tumbuka	751 (22.0)	347 (46.2)	404 (53.8)	
Lomwe	462 (13.5)	218 (47.2)	244 (52.8)	
Yao	358 (10.5)	152 (42.5)	206 (57.5)	
Ngoni	392 (11.5)	227 (57.9)	165 (42.1)	
Other	482 (14.0)	232 (48.4)	250 (51.6)	
Region				0.5540
Northern	1176 (34.2)	544 (46.3)	632 (53.7)	
Central	1160 (33.9)	558 (48.1)	602 (51.9)	
Southern	1086 (53.9)	500 (46.0)	582 (54.0)	
Knowledge^a^				<.0001
Low	972 (28.3)	335 (34.5)	637 (65.5)	
Medium	748 (21.9)	337 (45.1)	411 (54.9)	
High	1702 (49.8)	930 (54.6)	772 (45.4)	

^a^Categorized based on tertiles

### Causes, symptoms and preventive characteristics of malaria by place of residence

The majority of women aged 15–49 years recognized fever (77.38%) as symptom of malaria, 97.17% reported mosquito bites as causes of malaria, and 96.93% said mosquito nets are a prevention method. Comprehensive results of all the characteristics that were used to assess the causes, symptoms and preventive measures of malaria are shown in Table 2.

**Table 2:** Causes, symptoms, and preventive characteristics by place of residence *n *= 3422. MMIS 2017

	Overall	Urban	Rural
	*n* (%)	*n* (%)	*n* (%)
Causes of malaria			
Mosquito bites (Yes)	3325 (97.2)	1576 (98.4)	1749 (96.1)
Eating immature sugarcane (No)	3414 (99.8)	1596 (99.6)	3414 (99.9)
Eating cold Nsima (No)	3404 (99.5)	1593 (99.4)	1811 (99.5)
Eating dirty food (No)	3333 (97.4)	1564 (97.6)	1769 (97.2)
Drinking dirty water (No)	3326 (97.3)	1563 (97.6)	11,766 (97.0)
Getting soaked in rain (No)	3365 (98.3)	1561 (97.4)	1804 (99.1)
Cold or changing weather (No)	3292 (96.2)	1545 (46.9)	1747 (53.1)
Symptoms of malaria			
Fever (Yes)	2648 (77.4)	1270 (79.3)	1378 (75.7)
Feeling cold (Yes)	1762 (51.5)	779 (48.6)	983 (54.0)
Headache (Yes)	2214 (64.7)	955 (59.6)	1259 (69.2)
Nausea and vomiting (Yes)	1874 (54.8)	902 (56.3)	972 (53.4)
Diarrhea (Yes)	2589 (75.7)	1146 (71.5)	1443 (79.3)
Dizziness (Yes)	3335 (97.5)	1548 (96.6)	1787 (98.2)
Loss of appetite (Yes)	3192 (93.3)	1435 (89.6)	1757 (96.5)
Body ache or joint pain (Yes)	2029 (59.3)	918 (57.3)	1111 (61.0)
Pale eyes (Yes)	3342 (97.7)	1570 (98.0)	1772 (97.4)
Salty-tasting palms (Yes)	3413 (99.7)	1595 (99.6)	1818 (99.9)
Feeling weak (Yes)	3011 (88.0)	1390 (86.8)	1321 (89.1)
Refuse to eat or drink (Yes)	3357 (98.1)	1579 (986)	1778 (97.7)
Prevention against malaria			
Sleep under a treated net (Yes)	2209 (64.6)	1037 (64.7)	1172 (64.4)
Sleep under an ITN (Yes)	1150 (33.6)	568 (35.5)	582 (32.0)
Use mosquito net (Yes)	3317 (96.9)	1510 (94.3)	1807 (99.3)
Take preventive medication (Yes)	139 (4.1)	67 (4.2)	72 (4.0)
Spray the house/rooms with insecticide (Yes)	143 (4.2)	110 (6.9)	33 (1.8)
Clear weeds around the house (Yes)	356 (10.4)	168 (10.5)	188 (10.3)
Use mosquito coils (Yes)	3290 (96.1)	1502 (93.7)	1788 (98.2)
Cut grass around house (Yes)	507 (14.8)	264 (16.5)	243 (13.4)
Fill in stagnant waters (puddles) (Yes)	758 (22.2)	432 (27.0)	326 (17.9)
Keep surroundings clean (Yes)	923 (27.0)	426 (26.6)	497 (27.3)
Burn leaves (Yes)	27 (0.8)	11 (0.7)	16 (0.9)
Avoid drinking dirty water (No)	3272 (95.6)	1554 (97.0)	1718 (94.4)
Avoid eating bad food (No)	3342 (97.7)	1574 (98.3)	1768 (97.1)
Put screens on windows (Yes)	7 (0.2)	6 (0.4)	1 (0.1)
Avoid getting soaked in rain (No)	3395 (99.2)	1585(99.9)	1810 (99.5)

*ITN* insecticide-treated nets

### Characteristics of the study participants by levels of knowledge

Table 3 displays the prevalence of levels of knowledge related to the causes, signs and symptoms, and preventive measures of malaria among women of reproductive age by selected characteristics. The levels of knowledge related to the causes, signs and symptoms, and preventive measures of malaria was significantly high among women of age group 40–49 years (*P *< .0001), among women with secondary and higher education (*P *< .0001), and among women who had a radio (*P *< .0001) as well as those who had television in their households (*P *< *.0001*). Furthermore, the high levels of knowledge related to the causes, signs and symptoms, and preventive measures of malaria was observed among women from richest households (*P *< .0001), among women who seen/heard about malaria message (*P *< .0001), among Tumbuka women (*P *< .0001), among urban (*P *< *.0001*) and northern dwellers (*P *= *0.0001*).

**Table 3:** Characteristics of study respondents by malaria knowledge^a^ among women. MMIS 2017

	Low	Medium	High	*P*-value
	*n* (%)	*n* (%)	*n* (%)	
Age of the respondents				0.0002
15–19	242 (34.4)	160 (22.8)	301 (42.8)	
20–24	205 (29.5)	154 (22.2)	335 (48.3)	
25–29	152 (25.8)	142 (24.1)	296 (50.1)	
30–34	151 (26.2)	128 (22.3)	296 (51.5)	
35–39	106 (24.3)	94 (21.6)	236 (54.1)	
40–49	116 (27.4)	70 (16.5)	238 (56.1)	
Highest educational level				<.0001
No formal education	144 (45.3)	56 (22.2)	82 (32.5)	
Primary	613 (33.2)	419 (22.7)	815 (44.1)	
Secondary and above	245 (18.5)	273 (20.6)	805 (60.9)	
Household has radio				<.0001
No	620 (34.2)	408 (22.6)	777 (43.2)	
Yes	352 (21.8)	340 (21.0)	925 (57.2)	
Household has television				<.0001
No	807 (33.1)	548 (22.4)	1087 (44.5)	
Yes	165 (16.9)	200 (20.4)	615 (62.8)	
Sex of household head				0.9604
Male	717 (28.5)	550 (21.9)	1247 (49.6)	
Female	255 (28.1)	198 (21.8)	455 (50.1)	
Wealth index				<.0001
Poorest/Poorer/Middle	463 (39.6)	275 (23.5)	431 (36.9)	
Richer	183 (29.2)	136 (21.7)	308 (49.1)	
Richest	326 (20.1)	337 (20.7)	963 (59.2)	
Seen/heard malaria message				<.0001
No	717 (32.6)	497 (22.6)	986 (44.8)	
Yes	255 (20.9)	251 (20.5)	716 (58.6)	
Ethnicity				<.0001
Chewa	327 (33.5)	219 (22.4)	431 (44.1)	
Tumbuka	189 (25.2)	138 (18.4)	424 (56.5)	
Lomwe	116 (25.1)	115 (24.9)	231 (50.0)	
Yao	133 (37.2)	84 (23.5)	141 (39.4)	
Ngoni	89 (22.7)	94 (24.0)	209 (53.3)	
Other	118 (24.5)	98 (20.3)	266 (55.2)	
Place of residence				<.0001
Urban	335 (20.9)	337 (21.0)	930 (58.1)	
Rural	637 (35.0)	411 (22.6)	772 (42.4)	
Region				0.0001
Northern	294 (25.0)	233 (19.8)	649 (55.2)	
Central	358 (30.9)	253 (21.8)	549 (47.3)	
Southern	320 (29.5)	262 (24.1)	504 (46.4)	

^a^Categorized based on the variable tertiles

### Characteristics of the study participants by educational level

Table 4 shows the characteristics of study respondents by highest educational level among women. Women who had secondary and higher education were more likely to own a radio (*P *< .0001) and television in their households (*P *< .0001). Furthermore, women who had secondary and higher education resided in richest households (*P *< .0001), had seen or heard malaria message (*P *< .0001), were from Tumbuka ethnic group (*P *< .0001), were urban (*P *< .0001) and northern region (*P *< .0001) dwellers.

**Table 4:** Characteristics of study respondents by highest educational level among women. MMIS 2017

Characteristics	No education	Primary education	Secondary and above education	*P*-value
	*n* (%)	*n* (%)	*n* (%)	
Age of the respondents				<.0001
15–19	13 (1.85)	400 (56.90)	290 (41.25)	
20–24	29 (4.18)	336 (48.41)	329 (47.41)	
25–29	29 (4.92)	308 (52.20)	253 (42.88)	
30–34	43 (7.48)	311 (54.09)	221 (38.43)	
35–39	44 (10.09)	253 (58.03)	139 (31.88)	
40–49	94 (22.17)	239 (56.37)	91 (21.46)	
Household has radio				<.0001
No	182 (72.22)	1145 (61.99)	478 (36.13)	
Yes	70 (27.78)	702 (38.01)	845 (63.87)	
Household has television				<.0001
No	240 (95.24)	1573 (85.17)	629 (47.54)	
Yes	12 (4.76)	274 (14.83)	694 (52.46)	
Wealth index^†^				<.0001
Poorest/Poorer/Middle	167 (66.27)	866 (46.89)	136 (10.28)	
Richer	52 (20.63)	403 (21.82)	172 (13.00)	
Richest	33 (13.10)	578 (31.29)	1015 (76.72)	
Seen/heard malaria message				<.0001
No	207 (82.14)	1293 (70.01)	700 (52.91)	
Yes	45 (17.86)	554 (29.99)	623 (47.09)	
Ethnicity				<.0001
Chewa	104 (41.27)	538 (29.13)	335 (25.32)	
Tumbuka	14 (5.56)	363 (19.65)	374 (28.27)	
Lomwe	28 (11.11)	270 (14.62)	164 (12.40)	
Yao	43 (17.06)	224 (12.13)	91 (6.88)	
Ngoni	25 (9.92)	194 (10.50)	173 (13.08)	
Other	38 (15.08)	258 (13.97)	186 (14.06)	
Place of residence				<.0001
Urban	52 (20.63)	609 (32.97)	941 (71.13)	
Rural	200 (79.37)	1248 (67.03)	382 (28.87)	
Geographical region				<.0001
Northern	37 (14.68)	590 (31.94)	549 (41.50)	
Central	109 (43.25)	648 (35.09)	403 (30.46)	
Southern	106 (42.06)	609 (32.97)	371 (28.04)	

A composite measure of a household’s cumulative living standard. It is calculated by using easy-to-collect data on a household’s ownership of selected assets, such as televisions and bicycles; materials used for housing construction and types of water access and sanitation facilities

### Characteristics of the study participants by pregnancy status

Table 5 shows the characteristics of study respondents by pregnancy status. The prevalence of pregnancy was significantly different by age of women (*P *< .0001), educational level (*P *= 0.0315), possession of television (*P *= 0.0494), gender of household head (*P *< .0001), wealth index (*P *= 0.0042), malaria message (*P *= 0.0213), and place of residence (*P *= 0.0026).

**Table 5:** Characteristics of study respondents according to pregnancy status *n *= 3422. MMIS 2017

	Total sample	Non-pregnant	Pregnant	*P*-value
	*n* (%)	*n* (%)	*n* (%)	
Age of the respondents				<.0001
15–19	703 (20.5)	670 (20.9)	33 (15.0)	
20–24	694 (20.3)	620 (19.4)	74 (33.8)	
25–29	590 (17.2)	543 (17.0)	47 (21.5)	
30–34	575 (16.8)	530 (16.6)	45 (20.6)	
35–39	436 (12.7)	419 (13.1)	17 (7.8)	
40–49	424 (12.39)	421 (13.1)	3 (1.4)	
Highest educational level				0.0315
No formal education	252 (7.4)	244 (7.6)	8 (3.7)	
Primary	1847 (54.0)	1714 (53.5)	133 (60.7)	
Secondary and above	1323 (38.7)	1245 (38.9)	78 (35.6)	
Household has radio				0.6259
No	1805 (52.75)	1686 (52.64)	119 (54.3)	
Yes	1617 (47.3)	1517 (47.3)	100 (45.7)	
Household has television				0.0494
No	2442 (71.4)	2273 (71.0)	169 (77.2)	
Yes	980 (28.6)	930 (29.0)	50 (22.8)	
Sex of household head				<.0001
Male	2514 (73.5)	2327 (72.7)	187 (85.4)	
Female	908 (26.5)	876 (27.3)	32 (14.6)	
Wealth index				0.0042
Poorest/Poorer/Middle	1169 (34.2)	1073 (33.5)	96 (43.8)	
Richer	627 (18.3)	587 (18.3)	40 (18.3)	
Richest	1626 (47.5)	1543 (48.2)	83 (37.9)	
Seen/heard malaria message				0.0213
No	2200 (64.3)	2075 (64.8)	125 (57.1)	
Yes	1222 (35.7)	1128 (35.2)	94 (42.9)	
Ethnicity				0.6892
Chewa	977 (28.6)	909 (28.4)	68 (31.1)	
Tumbuka	751 (22.0)	703 (22.0)	48 (21.9)	
Lomwe	462 (13.5)	428 (13.4)	34 (15.5)	
Yao	358 (10.5)	336 (10.5)	22 (10.1)	
Ngoni	392 (11.5)	369 (11.5)	23 (10.5)	
Other	482 (14.1)	458 (14.3)	24 (11.0)	
Place of residence				0.0026
Urban	1602 (46.8)	1521 (47.5)	81 (37.0)	
Rural	1820 (56.2)	1682 (52.5)	138 (63.)	
Region				0.6803
Northern	1176 (34.4)	1105 (34.5)	71 (32.4)	
Central	1160 (33.9)	1080 (33.7)	80 (36.5)	
Southern	1086 (54.0)	1018 (31.8)	68 (31.1)	
Knowledge^a^				0.7980
Low	979 (28.6)	915 (28.6)	64 (29.2)	
Medium	1358 (39.7)	1268 (39.6)	90 (41.1)	
High	1085 (31.7)	1020 (31.9)	65 (29.7)	

^a^Categorized based on tertiles

### Factors associated with the levels of knowledge related to the causes, symptoms and preventive measures of malaria: low *versus* high knowledge

Table 6 presents the factors associated with the levels of knowledge related to the causes, signs and symptoms, and preventive measures of malaria among adult women. Compared to women aged between 40 and 49 years, younger women (15–19 years) had high odds of having low knowledge (aOR: 2.58; 95% CI 1.69–3.92); *P *< .0001. Moreover, the odds of low knowledge were also significantly high among women who had no formal education (aOR: 3.73; 95% CI 2.20–6.33); *P *< .0001 compared to women who had secondary and higher education. Furthermore, the odds of having low levels of knowledge related to the causes, signs and symptoms, and preventive measures of malaria were high in women who had no television in their households (aOR: 1.50; 95% CI 1.02–2.22); *P *= 0.0393 as well as women who had not seen or heard malaria messages (aOR: 1.53; 95% CI 1.20–1.95); *P *= 0.0008 compared to women who had television in their households and had seen or heard malaria message. Additionally, compared to women from other ethnic groups, Yao women had high odds of having low knowledge (aOR: 1.95; 95% CI 1.10–3.46); *P *= 0.0219 related to the causes, signs and symptoms, and preventive measures of malaria. Conversely, the women from urban areas were 43% (aOR: 0.57; 95% CI 0.36–0.92); *P *= 0.0228 less likely to have low knowledge. Table 6 shows the effect of selected factors associated with the knowledge score of the causes, symptoms and preventive measures of malaria. The results showed that women aged 15–19 years (beta [β] =  − 0.73, standard error [SE] = 0.12); *P *< .0001, women with no formal education (β =  − 1.17, SE = 0.15); *P *< .0001, women whose household had no radio (β = − 0.15, SE = 0.08); *P *= 0.0715 and women who had not seen or heard malaria message (β = − 0.41, SE = 0.07); *P *< .0001 were negatively associated with high knowledge score.

**Table 6:** Multinomial adjusted odds ratios and multiple linear regression of malaria related knowledge^a^ among the adult women in Malawi

	Low vs high knowledge	*P*-value	Medium vs high knowledge	*P*-value	Knowledge score^c^	*P*-value
	AOR 95% (CI)		AOR 95% (CI)		β (SE)	
Maternal age (years)						
15–19	2.58 (1.69–3.92)	<.0001	2.03 (1.37–3.03)	0.0006	− 0.73 (0.12)	<.0001
20–24	1.84 (1.19–2.84)	0.0064	1.78 (1.15–2.78)	0.0107	− 0.61 (0.12)	<.0001
25–29	1.45 (0.96–2.21)	0.0791	1.92 (1.30–2.84)	0.0012	− 0.39 (0.12)	0.0017
30–34	1.29 (0.82–2.03)	0.2713	1.48 (0.95–2.29)	0.0816	− 0.27 (0.12)	0.0300
35–39	1.17 (0.79–1.74)	0.4382	1.39 (0.83–2.38)	0.2031	− 0.24 (0.13)	0.0728
40–49	1.00		1.00		1.00	
Highest educational level						
No education	3.73 (2.20–6.33)	<.0001	1.98 (1.25–3.14)	0.0041	− 1.17 (0.15)	<.0001
Primary	2.02 (1.51–2.70)	<.0001	1.39 (1.04–1.86)	0.0274	− 0.65 (0.08)	<.0001
Secondary and above	1.00		1.00		1.00	
Household has radio						
No	1.14 (0.85–1.54)	0.3845	0.99 (0.76–1.28)	0.9271	− 0.15 (0.0816)	0.0715
Yes	1.00		1.00		1.00	
Household has television						
No	1.50 (1.02–2.22)	0.0393	1.079 (0.75–1.56)	0.6830	− 0.12 (0.1035)	0.2578
Yes	1.00		1.00		1.00	
Wealth index^b^						
Poorest/Poorer/Middle	1.20 (0.70–2.04)	0.5078	1.101 (0.68–1.78)	0.6919	− 0.14 (0.1371)	0.2995
Richer	0.87 (0.56–1.35)	0.5266	0.916 (0.58–1.44)	0.7024	− 0.02 (0.1217)	0.8562
Richest	1.00		1.00		1.00	
Seen/heard malaria message						
No	1.53 (1.20–1.95)	0.0008	1.23 (0.92–1.66)	0.1667	− 0.41 (0.07)	<.0001
Yes	1.00		1.00		1.00	
Ethnicity						
Chewa	1.51 (0.90–2.54)	0.1212	1.14 (0.70–1.874)	0.5953	− 0.10 (0.14)	0.4465
Tumbuka	0.84 (0.52–1.37)	0.4876	0.94 (0.56–1.595)	0.8266	− 0.05 (0.14)	0.7381
Lomwe	0.94 (0.56–1.60)	0.9619	1.19 (0.76–1.864)	0.4505	0.03 (0.15)	0.8256
Yao	1.95 (1.10–3.46)	0.0219	1.30 (0.81–2.069)	0.2695	0.01 (0.17)	0.9700
Ngoni	0.85 (0.47–1.54)	0.5937	0.99 (0.63–1.56)	0.9604	− 0.05 (0.16)	0.7407
Other	1.00		1.00		1.00	
Place of residence						
Urban	0.57 (0.36–0.92)	0.0228	0.64 (0.41–0.93)	0.0205	0.22 (0.17)	0.1780
Rural	1.00		1.00		1.00	
Geographical region						
Northern	0.77 (0.50–1.17)	0.2175	0.63 (0.40–0.99)	0.0438	0.30 (0.20)	0.1297
Central	0.87 (0.56–1.34)	0.5173	0.80 (0.55–1.16)	0.2365	0.04 (0.19)	0.8091
Southern	1.00		1.00		1.00	

*β* beta, *SE* standard error

^a^Categorized based on the variable tertiles; ^b^a composite measure of a household’s cumulative living standard. It is calculated by using easy-to-collect data on a household’s ownership of selected assets, such as televisions and bicycles; materials used for housing construction and types of water access and sanitation facilities; ^c^calculated as the sum of the 35 knowledge related variables, with 0 as the least possible score and 35 as highest possible score;

## Discussion

This is the first study to report the factors associated with knowledge score, also the levels of knowledge of the causes, symptoms, and prevention of malaria women of reproductive age in Malawi using a nationally representative sample in Malawi. Malaria KAP have reported the influence of malaria misconceptions on malaria control efforts [11, 16]. Often times, women play a vital role for their families in bringing about awareness regarding malaria prevention and control [29]. Thus, an effort to study the knowledge on causes, symptoms and preventive measures of malaria among women of reproductive was vital in appreciating the degree and influence of malaria programmatic efforts in malaria control in Malawi. Overall, the levels of knowledge in this study were reported to be moderate among adult women, which implies that the knowledge related to malaria should be scaled up.

Elsewhere lower levels of individual health knowledge have been reported to be linked with (i) increased rates of hospitalization and higher use of emergency care; (ii) poorer ability to demonstrate taking medications appropriately and poorer ability to interpret labels and health messages; (iii) lower use of mammography and lower uptake of the influenza vaccine; (iv) poorer knowledge among patients of their own disease or condition and many more [10, 30, 31]. After considering a number of independent factors, there were lower levels of knowledge about the causes, symptoms and preventive measures of malaria in women of age group 15–19 years, women with no formal education, women whose household had no television, women who had not seen or heard malaria message, women of Yao tribe and women from rural areas. Older women, with education, and those who had seen malaria messages had increased knowledge levels.

In this study, younger women (15–19 years) were more likely to have lower levels of knowledge about the causes, symptoms and preventive measures of malaria. The reasons behind this finding cannot be expressed explicitly. However, lack of experience among young mothers regarding childcare practices might contribute to the lower levels of knowledge related to malaria. Previous studies have also demonstrated that being inexperienced as a caregiver was one of the major risk factors of poor childhood care practices and poor health outcomes [32]. It can be hypothesized older women might have had prior malaria episodes, hence a high probability to have better knowledge about the causes, symptoms and prevention measures. Additionally, it is believed that older women might have had quite a lot of exposure to malaria messages compared to younger counterparts [33]. Furthermore, older women’s better knowledge could be related to their experiences during pregnancy [34].

Although insignificant, the finding indicated that women from the poorest households had low levels of malaria-related knowledge. This association may be due to the fact that women from the poorest households may find it hard to access information regarding malaria [35]. Poverty does not only encompasses low income but also shortage of resources, limited ability to meet basic needs (including access to information), and a wide range of other dimensions of vulnerability [36]. Furthermore, place of residence may be another factor to affect socially disadvantaged households. In settings like Malawi, place of residence (rural areas) may hinder women from the poorest households to access health information. Rural residents usually have barriers to healthcare that limit their ability to obtain the care they need, including access to health information and health literacy [17]. This is evidenced by the results presented in Table 1 where a majority of poorest women were from rural areas. Women are more likely to miss information related to malaria due to problems with distance to the nearest health facility as well as poor road networks to reach to the health facility. Previous studies also suggested that women from poor households may have barriers to access health services, including access to health information compared to mothers from richer households [37].

The current study showed that educational status was an important variable which was significantly associated with knowledge on malaria among women of reproductive age. The finding of this study are in line with the studies that were conducted in Nigeria [13], Burkina Faso [24] and Ghana [33]. An explanation of this association may be due to the fact that educated mothers may not have problems reading and comprehending information concerning malaria. Another reason might be that highly educated women may reside in richer households and urban areas where distance to health facilities may not be a problem [38]. Further, educated women may have better knowledge about their own health as well as better health-seeking behaviour [39–41]. This study has shown that women who had seen or heard malaria messages had better knowledge score and that women who had secondary and higher education were more likely to have seen or heard malaria messages. Furthermore, a large volume of literature has reported the positive association between health literacy and health-seeking behaviour, as well as behaviour related to malaria [42, 43]. Health literacy refers to personal characteristics and social resources required for people to access, read, understand and utilize information to make informed decisions about their health [44, 45]. Elsewhere it was reported that health literacy was a significant predictor of information-seeking preference in older people in general [43].

The findings of this study showed that living in the urban areas increased the level of knowledge on malaria among women. An explanation may be that women who are from urban areas may be more exposed for messages or information such as mass media and other health-related messages than women from rural areas, such as television, ratio, newspapers, posters or billboards, peer educators, etc. The current study results support this theory where women who had access to radio, television and had seen or heard malaria messages were more likely to be urban dwellers. This finding was also reported in prior studies done in Nigeria [18], Ethiopia [34], and Tanzania [19].

Finally, the finding of this study reported that Yao women were more likely to have lower knowledge of malaria. Usually, the differences in ethnicity reflect variations with respect to sociodemographic, economic and environment among ethnic groups. In Malawi, Yao tribe is known to have few people with formal education due to their cultural values. The current study’s findings demonstrated that women from Yao tribe were less likely to attain secondary and higher education and additionally, the level of education was associated with higher score for malaria preventive action similar to previous studies [19].

### Strengths and limitations

The study used nationally representative datasets; findings can be generalized to Malawian women. As this is the first study to evaluate knowledge levels regarding malaria, it may serve to inform public health programmes for the need to scale up efforts in improving malaria knowledge so as to complement other prevention strategies that are being implemented at national level. A number of issues were considered when evaluating knowledge levels, including causes, symptoms as well as prevention measures, providing more comprehensive information regarding malaria knowledge among Malawian women. However, this study is not without limitations. First, owing to the cross-sectional nature of the data, causal inferences in relationships of the selected characteristics and malaria knowledge could not be made. Second, measures concerning the women’s knowledge of the causes, symptoms and prevention measures of malaria and other behaviour were based on self-reports and there is possibility of recall bias. Third, there were no variables available to assess knowledge of malaria-related pregnancy outcomes.

## Conclusions

The current study demonstrated poor levels of knowledge among women of reproductive age, which highlights the need to upscale dissemination of knowledge related to malaria. Transmission of malaria can be best controlled by concentrating behavioural, tailored interventions so as to improve the knowledge of malaria especially for younger women, women with no formal education, women whose households have no media, women from Yao tribes and rural dwellers.

## Data Availability

The datasets generated and/or analyzed during the current study are available in the MEASURE DHS repository; https://dhsprogram.com/data/.

## Abbreviations

ACT: Artemisinin-based combination therapy
aORs: Adjusted odds ratios
CFR: Code of Federal Regulations
CIs: Confidence intervals
EAs: Enumeration areas
IPTp: Intermittent preventive treatment for pregnant women
IRB: Institutional Review Board
ITN: Insecticide-treated bed nets
KAP: Knowledge, attitudes, and practices
LBW: Low birth weight
MIS: Malawi Malaria Indicator Survey
MoH: Ministry of Health
NC: New York City
NHSRC: National Health Sciences Research Committee
NMCP: National Malaria Control Programme
PCA: Principal component analysis
PHC: Population and Housing Census
PTB: Preterm birth
RBM-MERG: Roll Back Malaria Monitoring and Evaluation Reference Group
SP: Sulfadoxine-pyrimethamine
SSA: Sub-Saharan Africa
USA: United States of America
VIF: Variance Inflation Factor
WHO: World Health Organization
